# Comparative genomics between human and animal associated subspecies of the *Mycobacterium avium* complex: a basis for pathogenicity

**DOI:** 10.1186/s12864-015-1889-2

**Published:** 2015-09-15

**Authors:** Verlaine J. Timms, Karl A. Hassan, Hazel M. Mitchell, Brett A. Neilan

**Affiliations:** School of Biotechnology and Biomolecular Sciences, University of New South Wales, Sydney, 2052 Australia; Department of Chemistry and Biomolecular Sciences, Macquarie University, Sydney, Australia; Centre for Infectious Diseases and Microbiology, Institute of Clinical Microbiology and Medical Research, Westmead Hospital, Sydney, NSW Australia

**Keywords:** *Mycobacterium avium*, *Mycobacterium paratuberculosis*, Genome analysis, Pathogenicity, PE/PPE family, Mycobactin, Mammalian cell entry, Inflammatory bowel disease, Johne’s disease, Host-pathogen interactions

## Abstract

**Background:**

A human isolate of *Mycobacterium avium* subsp. *paratuberculosis* (*M. paratuberculosis* 43525) was sequenced and compared genomically to other mycobacterial pathogens. *M. paratuberculosis* 43525 was recently isolated from a patient with ulcerative colitis and belongs to the *M. avium* complex, a group known to infect both humans and animals. While *M. paratuberculosis* is a known pathogen of livestock, there are only 20 human isolates from the last 20 years, therefore we took the opportunity to perform a whole genome comparison between human and animal mycobacterial pathogens. We also compared virulence determinants such as the mycobactin cluster, PE/PPE genes and mammalian cell entry (*mce*) operons between MAC subspecies that infect animals and those that infect humans. *M. tuberculosis* was also included in these analyses given its predominant role as a human pathogen.

**Results:**

This genome comparison showed the PE/PPE profile of *M. paratuberculosis* 43525 to be largely the same as other *M. paratuberculosis* isolates, except that it had one PPE and one PE_PGRS protein that are only present in human MAC strains and *M. tuberculosis*. PE/PPE proteins that were unique to *M. paratuberculosis* 43525, *M. avium* subsp. *hominissuis* and a caprine *M. paratuberculosis* isolate, were also identified. In addition, the mycobactin cluster differed between human and animal isolates and a unique *mce* operon flanked by two mycobactin genes, *mbtA* and *mbtJ*, was identified in all available *M. paratuberculosis* genomes.

**Conclusions:**

Despite the whole genome comparison placing *M. paratuberculosis* 43525 as closely related to bovine *M. paratuberculosis*, key virulence factors were similar to human mycobacterial pathogens. This study highlights key factors of mycobacterial pathogenesis in humans and forms the basis for future functional studies.

**Electronic supplementary material:**

The online version of this article (doi:10.1186/s12864-015-1889-2) contains supplementary material, which is available to authorized users.

## Background

*M. avium* subsp. *paratuberculosis* (*M. paratuberculosis*), of the *M. avium* complex (MAC), is one of the slowest growing mycobacteria and like other pathogenic mycobacteria, is difficult to detect and treat. It is widely recognised as the cause of Johne’s disease, a gastrointestinal disease of livestock, and is also implicated in human Crohn’s disease [1–3]. The MAC contain subspecies that infect animals and subspecies pathogenic to humans [4]. The closely related MAC display slight genomic differences depending on their host and comparison of these differences has the potential to identify host specific pathogenicity factors, leading to improved diagnosis and treatment.

In the current study we compared the genome of a newly isolated strain of *M. paratuberculosis* (*M. paratuberculosis* 43525) from a female patient with ulcerative colitis [5], to other pathogenic mycobacteria using Single Nucleotide Polymorphism (SNP) analysis, BLASTp (homology based) and phmmer (non-homology based) algorithms. While *M. paratuberculosis* normally infects animals, its isolation from humans is rare, with less than 20 isolates reported in the last 20 years [6–9]. Like other *M. paratuberculosis* from humans, *M. paratuberculosis* 43525 is cattle type (C-Type) [5]. Therefore, further analysis of this strain provides a unique opportunity to explore other possible variations in host pathogenicity factors.

While genomic studies have compared a number of these isolates to other *M. paratuberculosis* strains and *M. hominissuis* [10–15], all sequences of *M. paratuberculosis* to date have been obtained from laboratory strains of unknown subculture number, and often have undergone many years of laboratory passage. Current evidence would suggest that multiple subculture of *M. tuberculosis* may affect virulence properties with, for example, marked changes in cell wall lipids observed after extensive laboratory passage [16]. Important to note is that *M. bovis* BCG, widely used in vaccines due to its attenuation in immunocompetent hosts, was produced by multiple subculture in vitro [17]. In contrast, the genome of *M. paratuberculosis* 43525 was sequenced after only four subcultures and therefore provides a more accurate representation of the wild-type in vivo mycobacterial genome.

The virulence factors explored in this study, the PE/PPE (proline-glutamate/proline-proline-glutamate motif) genes, mammalian cell entry (*mce*) operons and the mycobactin cluster, were chosen based on studies into *M. tuberculosis* and *M. avium* pathogenicity. The analysis of these genomic loci afford the representation of pathogenicity elements present in *M. tuberculosis* isolated from human infections and *M. paratuberculosis* isolated from livestock infections [4, 18–22].

The PE/PPE families are unique to mycobacteria and were first identified for their ability to stimulate IFN-γ [19]. They are GC rich and thought to be the main source of strain variability within the MAC [4]. PE and PPE refer to the residues, Proline-Glutamate and Proline-Proline-Glutamate, respectively, located at the N termini of their encoded proteins. The *M. tuberculosis* genome devotes 10 % of its protein coding potential to this protein family with various functions attributed to them [23]. Similar to *M. tuberculosis*, some PPE of *M. paratuberculosis* are expressed on the cell surface, while others are cell wall associated and interact with the immune system via TLR-2 [19], however, this gene family only represents 2.5 % of the *M. paratuberculosis* genome [24].

The mammalian cell entry (*mce*) operons of *M. tuberculosis* were first discovered in studies to elucidate how *M. tuberculosis* enters non-phagocytic cells [25]. The genes exist in many bacterial species, however, only in the mycobacteria do they exist as operons [26]. The function of these operons is now thought to be diverse and not confined to cell entry, given that they have been found in non-pathogenic, environmental mycobacteria [26]. There are four *mce* operons in *M. tuberculosis*, each has two *yrbE* genes and six *mce* genes, often coupled to a *mce* regulator gene. The genes in each operon are contiguous but differentially expressed, depending on growth conditions and/or nutrient supply [21]. In the MAC, additional *mce* operons have been reported that do not appear to have orthologues in *M. tuberculosis* [26, 18].

Mycobactin dependency in vitro is a major phenotypic difference between *M. paratuberculosis* and other subspecies of the MAC complex. Mycobactins are siderophores that transport or scavenge iron, particularly in environments where free iron is limited, such as inside a host cell [27]. Like other siderophores, mycobactin is a secondary metabolite, a product of non-ribosomal peptide synthases (NRPS) and polyketide synthases (PKS) (an integrated NRPS-PKS) [28]. The mycobactin gene cluster contains 10 genes (A-J) and the mycobactin operon promoter is active in *M. paratuberculosis*, with all mycobactin genes able to be transcribed inside bovine macrophages [29]. *M. paratuberculosis* 43525 has a peculiar mycobactin phenotype as it grows on some types of media, such as Middlebrook 7H10, without the addition of mycobactin [5]. Given this, comparison of *M. paratuberculosis* 43525 with other MAC strains has the potential to provide unique genomic information and the basis for their pathogenicity.

## Results

The general features of the assembled draft genome of *M. paratuberculosis* 43525 are presented in Table 1. Out of a total of 4433 protein coding sequences (CDS), 1517 (35 %) belonged to recognised subsystems. Of the 2781 non-hypothetical CDS, 1450 belonged to recognised subsystems while 1715 CDS were hypothetical and of these, 67 belonged to subsystems according to RAST.

**Table 1:** Chromosome features of *M. paratuberculosis* 43525

Parameters	*M. paratuberculosis* 43525
Reference organism	*M. paratuberculosis* K10
Chromosome size (base pairs)	4,812,039
G + C (%)	69.7
Number of contigs	466
Protein-coding sequences (CDS)	4433
CDS belonging to subsystems	1517
Non-hypothetical CDS	2781
Non-hypothetical CDS belonging to subsytems	1450
Hypothetical CDS	1715
Hypothetical CDS belonging to subsystems	67
Number of Subsystems	372
No. of RNAs	49

**Fig. 1:**
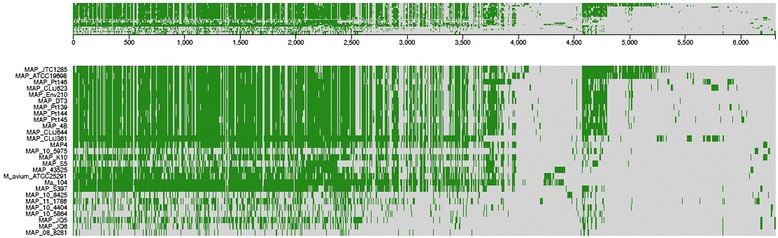
Graphical representation of the 6310 orthologous clusters of annotated protein sequences encoded in 27 mycobacterial strains. Comparative blastp 2.2.22+ searches [61] were conducted using Proteinortho [30] and orthologous clusters were visualised using FriPan (http://www.vicbioinformatics.com/software.fripan.shtml)

Comparative blastp searches and clustering analyses executed through Proteinortho [30], suggested that 165 putative protein sequences annotated in the *M. paratuberculosis* 43525 genome were unique to this strain (Fig. 1). These putatively unique sequences included a large number of hypothetical proteins, as well as PE-PGRS and mce genes that will be described below. In addition, differences were observed between the genes encoding the mycobactin cluster and this cluster was analysed in more detail.

### Single nucleotide polymorphism (SNP) analysis

**Fig. 2:**
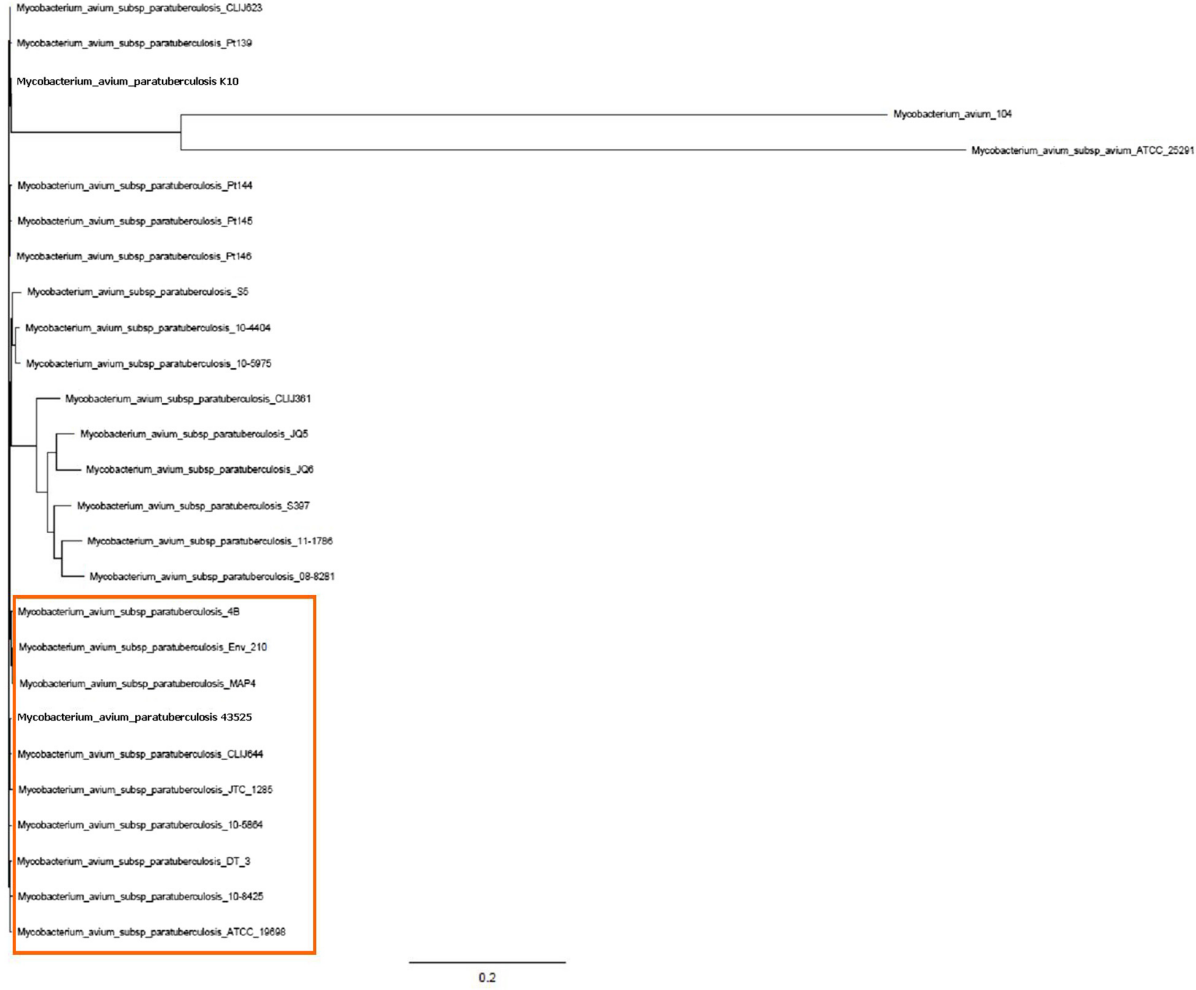
Phylogenetic analysis of *M. paratuberculosis* 43525 relative to other *M. paratuberculosis* isolates and two *M. avium* isolates. Neighbour-joining phylogenetic tree based on SNPs of *M. paratuberculosis* isolates from bovine, ovine and human hosts and *M. avium* isolates derived from human and avian hosts with *M. paratuberculosis* K10 as the reference. The *colour box* indicates the clade in which *M. paratuberculosis* 43525 belongs and also contains human isolates *M. paratuberculosis* 4 and 4B. In addition, this clade contains recent (compared to the type strains) bovine isolates, including the bovine isolate CLIJ644. The reference strain *M. paratuberculosis* K10 is in a different clade to *M. paratuberculosis* 43525 and the type strain ATCC19698

To better characterise *M. paratuberculosis* 43525, variation between this bacterium and 27 other mycobacterial strains (including two *M. avium* subsp. *avium* strains) were compared at the nucleotide level. Of these strains, nine were *M. paratuberculosis* isolates from humans, one was an ovine isolate and one was a caprine isolate. The SNPs of all strains were concatenated and used for phylogenetic analysis on a genome-wide level with *M. paratuberculosis* K10 as the reference strain. The rooted tree (Fig. 2) shows *M. paratuberculosis* 43525 to be closely related to *M. paratuberculosis* CLIJ644, a bovine isolate from Victoria, Australia [12].

### PE/PPE genes

**Fig. 3:**
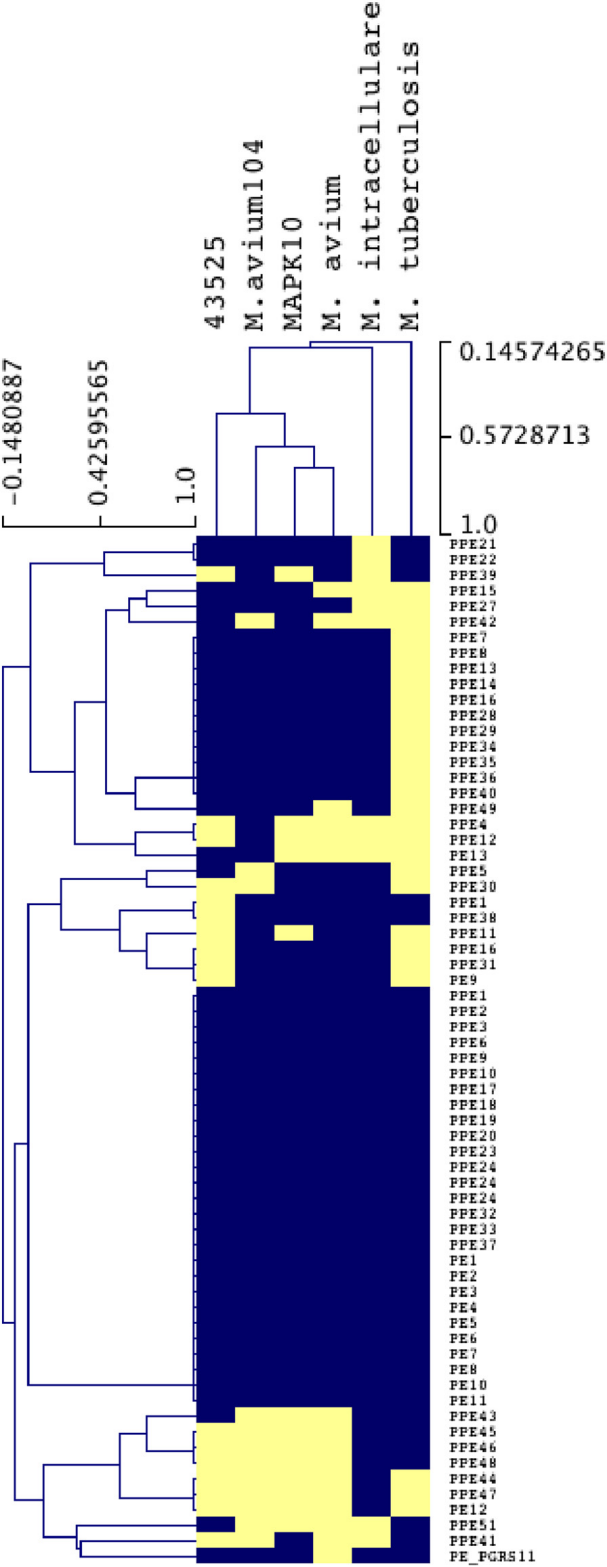
A summary of the presence and absence of the MACPE/PPE. The genes were sorted according to their distribution profiles (Additional file 1). Orthologues in *M. tuberculosis* were also added for comparison. *Blue* indicates the gene (listed on the *right hand side*) is present while *yellow* indicates the gene is missing. The strain order across the *top* is determined by the relative presence/absence of PE/PPE genes

The nomenclature of the MAC complex PE/PPE genes was used as previously described [4], and a summary of the *M. paratuberculosis* 43525 genes shared between the MAC and *M. tuberculosis* is presented in Fig. 3 and Additional file 1. Thirty seven PPE genes were found in *M. paratuberculosis* 43525, none of which were unique to this strain, while 17 were conserved in all strains examined. In the bovine strain *M. paratuberculosis* K10, MACPPE15 is a fragmented pseudogene, whereas the full gene is present in the human isolate *M. paratuberculosis* 43525, and this gene is homologous to Mav2514 from *M. avium* 104. Although MACPPE41 and MACPPE42 are said to be unique to the *M. paratuberculosis* subspecies, here only MACPPE42 was found in *M. paratuberculosis* 43525 [4].

Ten PE genes and one PE fragment were present in *M. paratuberculosis* 43525 (Fig. 3, Additional file 1). PE13 was the only PE gene that was not conserved in all strains studied, being found only in *M. paratuberculosis* 43525 and *M. avium* 104. The genome of *M. paratuberculosis* 43525 also had gene PE_PGRS11 which was also found in *M. paratuberculosis* K10, *M. tuberculosis* and *M. avium* 104, but absent in *M. avium* ATCC25291.

### Mycobactin

**Fig. 4:**
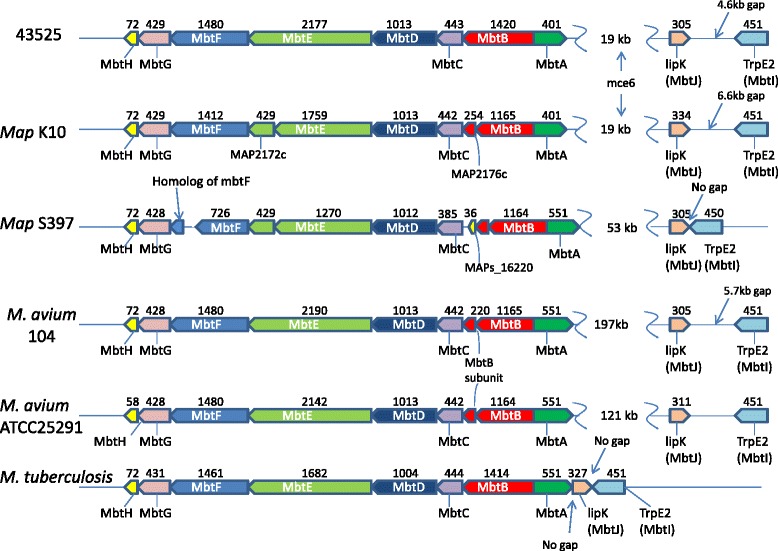
Comparison of the mycobactin cluster between members of the MAC complex and *M. tuberculosis*. Amino acid length is indicated above each gene, gene names are indicated and colour matched to equivalent genes in other strains (*M. paratuberculosis* 43525, *M. paratuberculosis* K10, *M. paratuberculosis* S397 *M. avium* 104, *M. avium* ATCC25291 and *M. tuberculosis*). *M. paratuberculosis* K10, *M. paratuberculosis* S397 and *M. avium* ATCC25291 are of animal origin, the remaining three are from human hosts. The NRPS modules MbtB and MbtE are one gene in *M. paratuberculosis* 43525 and *M. tuberculosis* but two genes in other MAC. In addition, all *M. paratuberculosis* strains had an mce operon (*mce*6) present in the gap between MbtA and MbtJ (indicated for *M. paratuberculosis* 43525 and K10)

A total of 17 NRPS/ PKS clusters were identified by antiSMASH in the *M. paratuberculosis* 43525 genome. The cluster identified as the mycobactin cluster was analysed and found to have a different primary structure as compared with that of other MAC strains, with respect to the spacing of genes and gene size (Fig. 4). The mycobactin cluster of *M. paratuberculosis* has previously been shown to have both NRPS and PKS modules [24]. While the *mbtA*, *mbtC*, *mbtD*, *mbtG* and *mbtI* genes of the *M. paratuberculosis* 43525 mycobactin cluster were found to be identical to the equivalent genes in *M. paratuberculosis* K10, the remaining 5 genes (3 of which are NRPS modules) were found to encode larger proteins.

Furthermore, the *mbtB* gene, shown by others to be the first gene involved in mycobactin synthesis [31], encodes a polypeptide of 1420 amino acids in *M. paratuberculosis* 43525, which was larger than the *mbtB* gene product of strains such as *M. paratuberculosis* K10 and *M. avium* 104 but similar in size to the equivalent MbtB in *M. tuberculosis*. The polypeptide size difference between these strains was due to the thioesterase domain of MbtB being encoded on a separate gene in *M. paratuberculosis* K10 and other MAC, but not in *M. paratuberculosis* 43525 or *M. tuberculosis* (Fig. 4).

The size and organisation of genes encoding MbtE vary greatly across strains (Fig. 4). The AMP binding and peptidyl carrier protein (PCP) domains were encoded on the one *mbtE* gene in *M. paratuberculosis* 43525, however, in *M. paratuberculosis* K10 the AMP binding was split across MAP2172c and MAP2173c, while the PCP domain is encoded on MAP2172c. Confirmation by amplicon sequencing demonstrated a 187 nucleotide indel in *M. paratuberculosis* 43525 compared to *M. paratuberculosis* K10 (bases 2411868 to 2412055). The AMP binding domain is a 215 aa protein in *M. paratuberculosis* K10 as compared with a 394 residue protein in *M. paratuberculosis* 43525 and a 408 aa protein in *M. avium* 104. Anti-smash uses a number of databases to predict the substrate for each NRPS domain. The predicted substrate for MbtE of *M. paratuberculosis* 43525 is tyrosine. In contrast, there was no consensus on the predicted substrate for MbtE of *M. paratuberculosis* K10 and *M. avium* 104.

The *mbtF* gene in *M. paratuberculosis* 43525 encoded a longer protein than in the *M. paratuberculosis* K10 equivalent. The main difference was in the epimerisation domain that encodes a shorter polypeptide in *M. paratuberculosis* K10 (179 aa) as compared with *M. paratuberculosis* 43525 (299 aa), *M. avium* 104 (299 aa), *M. avium* ATCC25291 (299 aa) and *M. tuberculosis* (288 aa).

Five copies of *mbtH* were found in *M. paratuberculosis* 43525, four of which (*mbtH*_*1*, *mbtH*_*2*, *mbtH*_*3* and *phoP*) had 100 % sequence similarity with the equivalent genes in *M. paratuberculosis* K10. The *mbtH*_*3* gene was situated adjacent to the mycobactin cluster. However, the fifth *mbtH* gene of *M. paratuberculosis* 43525, adjacent to *pst*A was found to have an 85 % match to *mbtH*_*2* in K10 but 100 % sequence similarity with 18 other *mbtH* like genes including the D522_08303 gene in another *M. paratuberculosis* strain (S5) originally isolated from a goat, MAP4_2610 from *M. paratuberculosis* MAP4 (a human isolate), MAH_2060 of *M. avium* TH135 and gene OCQ_31530 in *M. intracellulare* (strain MOTT-64).

In addition to differences in the size of genes within the mycobactin gene cluster, there were also differences in the spacing between the genes and gene clusters. The gap between *mbtA* and *mbtJ* (*lipK*) was comparable between *M. paratuberculosis* K10 and *M. paratuberculosis* 43525 and contained a *mce* operon 8.7 kbp downstream from *mbtA*. However, the gap between *mbtJ* (*lipK*) and *mbtI* (*trpE2*) in *M. paratuberculosis* 43525 was 2 kb shorter compared with the 6.6 kb spacer region in *M. paratuberculosis* K10 (Fig. 4).

### *mce* genes

The *M. paratuberculosis* 43525 genome contained eight *mce* operons that encode 74 *mce* proteins, although not all are complete. The four best known *mce* operons identified in *M. tuberculosis* are labelled *1*–*4* in Table 2. Other identified *mce* operons include *mce 5*, *6*, and *7*. Based on these findings it is suggested to include the gene designation *mce 8*, an operon that was originally described as a duplicate of *mce 7*. However, *mce 8* has low nucleotide and amino acid sequence similarity (72 and 63 %, respectively), when compared to the existing *mce7* in *M. paratuberculosis* [26]. Table 2 shows the amino acid sequence similarity of the *mce* genes in *M. paratuberculosis* 43525 compared to equivalent genes in related bacteria of the MAC and *M. tuberculosis*. Of particular note is that in all *M. paratuberculosis* isolates included in this study, *mce6* was found 8.7 kb downstream from *mbtA* of the mycobactin cluster.

**Table 2:** Percentage amino acid sequence similarity of *mce* operons in 43525 compared to *M. paratuberculosis* K10 (MAP K10), *M. avium* 104, *M. avium* TH135, *M. intracellulare* and *M. tuberculosis*

	MAP K10	Mav 104	Mav TH135	Mav ATCC25291	*M. intracellulare*	*M. tuberculosis*	Remarks
Operon							
*mce1*	99–100	77–100	99–100	81–100	92–99	76–93	3 genes in 43525 are longer, *fadD5* (453aa longer than MAP3601), *yrbE1B* (40aa longer than MAP3603), *mce1E* (233aa longer than MAP3608 and MavATCC25291_4409
*mce2*	99–100	97–99	99–100	97–100^a^	62–78	72–91	*Mce2E* missing in *M. avium* ATCC25291
*mce3*	99–100^a^	60–100	99	98–99	84–96	50–62	*yrbE3B* missing in MAPK10
*mce4*	100	99–100^a^	99–100	99–100	93–99	81–95	Frameshift in *M. avium* 104 *Mce4F*
*mce5*	99–100^a^	99–100^a^	65–99^a^	90–99	85–99	–	Deletion at position 7862892 of MAPK10 results in truncation of MAP0762 and *M. avium* ATCC25291_0785 proteins by 241aa and 70aa respectively. Frameshift in Mav0951.
*mce6*	99–100	–	–	–	88–92	–	Another human MAP strain, MAP4 had 100 % a.a. homology to all genes of 43525 in this operon.
*mce7*	99–100	98–100	98–99	98–100	88–97	–	This operon also found in *M. marinum* (>69 % homology) and *M. abscessus*.
*mce8*	97–100	96–100	96–100	95–99	80–94	–	Frameshift in MAP K10 (MAP0116). Truncated protein in MavATCC25291_0099. 43525 is 100 % identical to sheep MAP strains S397 and S5 for all genes in this operon.

Each value is the range across each operon. No value indicates that the operon is missing in that species/strain. ^a^indicates one or two genes are missing in that operon relative to isolate 43525 (see remarks)

Four genes of the *mce1* operon were longer than the corresponding genes in *M. paratuberculosis* K10 and the same size as the corresponding genes of *M. paratuberculosis* MAP4 and other MAC. While the *mce2* operon was conserved among *M. paratuberculosis*, *M. avium* strains 104 and TH135, the *mce3R* gene appeared to be missing from *M. paratuberculosis* 43525 and other *M. paratuberculosis* strains. As reported by others, the *mce4a* gene was highly conserved across all mycobacteria [21].

Of particular note was the finding that the conserved hypothetical integral membrane protein *yrbe3B* was present in *M. paratuberculosis* 43525 but missing in *M. paratuberculosis* K10. Interestingly, *yrbe3B* has been found in a *M. paratuberculosis* strain (S397) isolated from sheep, *M. avium* 104, *M. avium* TH135, *M. avium* ATCC25291, *M. intracellulare* and *M. tuberculosis*.

## Discussion

Using comparative genomics a rare human isolate of *M. paratuberculosis* was compared to both animal and human pathogens of the MAC and *M. tuberculosis*. After broad analysis by Blast and SNP typing, this study focused on comparisons of PE/PPE genes, the mycobactin cluster and the *mce* operons, all of which are key virulence factors across the species examined.

When compared at the nucleotide level, *M. paratuberculosis* 43525 displayed a close relationship to a bovine isolate *M. paratuberculosis* CLIJ644 (Fig. 2). This requires further investigation, particularly as *M. paratuberculosis* is shed in the milk of infected cows even at the early subclinical stage and that *M. paratuberculosis* can survive pasteurisation [32].

As in prior work, it was found that the complement of PPE genes was variable across strains while the PE genes showed a high degree of conservation (Fig. 3 and Additional file 1) [4]. A possible human associated PPE, MACPPE43 was present in *M. paratuberculosis* 43525 and *M. intracellulare*, which was orthologous to Rv3621c (PPE65 of *M. tuberculosis*). In contrast, MACPPE43 was not present in any strains of animal origin, including other *M. paratuberculosis* isolates. In *M. tuberculosis*, this gene was not essential for in vitro growth but could be detected in *M. tuberculosis* H37Rv infected guinea pig lungs at 30 and 90 days post infection suggesting a critical function for this gene product in vivo [33, 34]. PE_PGRS11 was also found to be present in strains isolated from humans, as well as *M. paratuberculosis* K10 although its function too is unknown. Two PE/PPE genes, MACPPE51 and a Mav2927 orthologue, were only found in the new human isolate *M. paratuberculosis* 43525, *M. hominissuis* and a caprine isolate of *M. paratuberculosis*, S5. Given the significance of the PE/PPE family to virulence, through generation of antigenic variations, the functions of the PE/PPE identified here should be investigated further.

MACPPE42 is unique to *M. paratuberculosis* and is located on a Large Sequence Polymorphism (LSP)-14 [4, 35]. There is some evidence that LSPs are associated with the cellular immune response [36] and it is co-transcribed with the iron-regulated transporters (irt) A and B equivalents MAP3734-3735 in macrophages [35]. IrtA and B are thought to be involved in the trafficking of carboxymycobactin, which is secreted in contrast to cell wall associated mycobactin [37]. As proposed in a recent study, MACPPE42 may act as a signal transduction protein for the IrtA and B equivalents which in turn form a single ABC transporter for Fe-carboxymycobactin and iron assimilation via ferric iron reduction [35]. Structural studies demonstrating the similarity of *M. tuberculosis* PPE proteins to signal transduction molecules and the observation that some PE/PPE proteins are up-regulated during iron limitation and repressed by the regulator *ideR*, form the basis of the above proposal [38, 39]. In addition, the finding that *M. tuberculosis mbtB* mutants that are unable to synthesise mycobactin or carboxymycobactin, but have *irtAB* intact, can grow in the presence of exogenous Fe-carboxymycobactin [40], may explain how mycobactin dependent strains of *M. paratuberculosis* survive the hostile environment of the macrophage as well as the mycobactin independence of other strains in vitro (as long as 1 % ferric ammonium citrate is added) [41, 42]. An attenuated strain of *M. paratuberculosis* (strain 316FNOR1960) has lost two of the *irtA* and B orthologues (MAP3734c and MAP3735c) as part of the Large Variable Genomic Island-19 deletion [43]. This strain was used in early vaccine preparations and was extensively subcultured before attenuation on Dubos media with pyruvate [44]. *M. paratuberculosis* is usually maintained on media containing ferric ammonium citrate rather than pyruvate, as the two are antagonistic once mycobactin is added [45]. MACPPE42 was not part of the vGI-19 deletion in the attenuated strain.

*M. paratuberculosis* 43525 did not require additional mycobactin on Middlebrook agar, a phenotype that has been described before in *M. paratuberculosis* isolates from sheep [46]. Therefore the mycobactin cluster required closer scrutiny and was found to differ in its primary structure when compared to *M. paratuberculosis* K10.

The three NRPS domains of the mycobactin cluster were larger in *M. paratuberculosis* 43525 as compared to *M. paratuberculosis* K10 and encode larger proteins. The size of the *mbtE* gene varies greatly across strains mainly because the AMP binding domain is smaller in *M. paratuberculosis* K10 as compared to equivalent domains in *M. paratuberculosis* 43525 and *M. avium* 104. The increase in product size results in a predicted substrate of tyrosine for this domain, while there is no prediction consensus for the equivalent substrate in *M. paratuberculosis* K10. Like *mbtB*, the *mbtE* gene has been shown to be crucial in the biosynthesis of both mycobactin and carboxymycobactin, with disruption of this gene in *M. tuberculosis* resulting in the loss of mycobactin and carboxymycobactin production and a drastically reduced ability to grow on agar [47]. However, unlike the *mbtB* mutant, the *mbtE* mutant of *M. tuberculosis* is unable to grow on iron replete media [47, 27]. Given that iron availability in an infected macrophage is thought to fluctuate [48, 49], mutations in *mbtE* resulting in the loss of mycobactin and carboxymycobactin production would likely hamper the ability of the pathogen to adapt and persist in this environment.

While other NRPS domains were larger in the *M. paratuberculosis* 43525 mycobactin cluster, none of these resulted in different substrate predictions. Currently there is no complete consensus on the substrate for the equivalent *mbtE* gene in *M. tuberculosis*, although a recent study has obtained the soluble megasynthase components (including MbtE) by co-producing them with MbtH [28]. Although the growth requirements of *M. paratuberculosis* 43525 suggest that it does produce a functional mycobactin, a similar functional study is needed to confirm this hypothesis as well as determine the structure of the isolate *M. paratuberculosis* 43525 mycobactin and whether this differs to mycobactins produced by other *M. paratuberculosis* strains.

*M. paratuberculosis* 43525 did, however, have an additional *mbtH* gene compared to *M. paratuberculosis* K10, with orthologues of this gene present in 18 other mycobacterial strains, and all with 100 % amino acid sequence homology. The MbtH proteins are thought to play a vital role in mycobactin precursor biosynthesis [50, 28]. *In vitro*, the activity of NRPS adenylating enzymes is stimulated by the addition of MbtH and further they have been shown to act as activators and/or chaperones in the NRPS assembly line [51, 50]. This may explain the different mycobactin phenotype apparent in *M. paratuberculosis* 43525 given that several *mbtH*-like genes can functionally replace each other [52].

A surprising link between the mycobactin cluster and the *mce* operons was observed in this study. A *mce* operon (*mce6*) exists 8.7 kbp downstream from *mbtA* and upstream of *mbtJ* and *mbtI*. The *mce* are thought to be involved in transport particularly under nutrient deplete conditions and each operon can be expressed at different stages of the growth cycle in *M. tuberculosis* [53, 21]. The *mce* also have high amino acid homology with ABC transport permeases which exist 15 kbp downstream of the *M. tuberculosis* mycobactin cluster [35]. The significance of the close proximity of this operon to the mycobactin cluster is currently unknown, however, further work to determine if this operon is co-transcribed with the mycobactin cluster is currently underway.

Also of note, *mce3R* was missing from *M. paratuberculosis* 43525 and other *M. paratuberculosis* genomes, a finding that may explain why the *mce3* operon appears to be non-functional in this subspecies. *mce3R* belongs to the TetR family and controls the expression of genes involved in β-oxidation and lipid metabolism in *M. tuberculosis* in vitro [54]. In *M. tuberculosis*, *mce3* mutants have been shown to grow slower than in the wild-type, thus providing a possible explanation for the longer doubling time of *M. paratuberculosis* [55].

*M. paratuberculosis* 43525, along with sheep strains of *M. paratuberculosis*, *M. avium* 104, *M. avium* TH135, *M. avium* ATCC25291 and *M. intracellulare*, was found to have the *yrbE3B* orthologue, unlike *M. paratuberculosis* K10. The function of *yrbE3B* is largely unknown but it is thought to be the permease component of an ABC-type transport system involved in resistance to organic solvents [21]. As yet the individual functions of the *mce3* genes have not been determined due to the fact that generating mutants for the genes in question has been extremely difficult [56]. The variable gene composition of the *mce3* operon between MAC strains may allow further studies to be performed to elucidate these functions.

## Conclusions

This study investigated human specific virulence genes of the mycobacteria and explored differences in the PE/PPE, *mce* and mycobactin cluster present in animal and human isolates of the MAC complex. Although *M. paratuberculosis* has long been thought of as the poor cousin when it comes to scavenging iron, the current study has shown for the first time the presence of unique PPE and *mce* genes that are possibly involved in both mycobactin and carboxymycobactin synthesis. Strains exist that appear to have only one mechanism of sequestering iron and *M. paratuberculosis* strains that display differing phenotypes form the basis of future functional studies designed to elucidate how pathogenic mycobacteria survive for long periods inside the host cells.

Given that the *M. paratuberculosis* 43525 genome is now publicly available, investigation of a range of other virulence factors present in the Mycobacteria, including *mmp*, the *esx* secretion pathway and the fatty acid synthesis genes can be undertaken which would shed further light on the ability of specific mycobacterial strains to colonise and cause disease in different tissues of different hosts.

## Methods

### Bacterial growth and genome sequencing

*M. paratuberculosis* 43525, isolated from a female with ulcerative colitis in 2009 [5], was grown on a slope of Middlebrook 7H10 agar supplemented with 10 % oleic acid-albumin-dextrose-catalase (OADC) (Difco) and 2 μg/mL mycobactin J (Allied Monitor) for 3 months. DNA was extracted as previously reported and the concentration and quality of DNA was measured using a Nanodrop ND-1000 spectrophotometer (Nanodrop Technologies) [57]. The genome of *M. paratuberculosis* 43525 was sequenced, using an Illumina HiSeq sequencer with the TruSeq SBS v4 GA kit. Paired-end indexed libraries were prepared from purified DNA fragments of approximately 320 bp in length generating raw reads of 100 bp in length. Sequencing was performed at the Ramaciotti Centre for Gene Function Analysis, University of New South Wales (Sydney, Australia). The sequence reads were submitted to the Sequence Read Database (http://www.ncbi.nlm.nih.gov/sra) and the SRA study accession for the *M. paratuberculosis* 43525 genome sequence is SRP033522.

### Genome assembly

Read quality was controlled by FASTQC (Babraham Bioinformatics) (http://www.bioinformatics.bbsrc.ac.uk/projects/fastqc) using default values. Raw reads were filtered for quality (mean phred > 20) and trimmed 10 bp on each end using custom Perl scripts, reducing each read to 80 bp. Paired-reads were then used to estimate the genome size using the program khmerfreq (kmer = 17). The trimmed reads were then assembled using Velvet 1.0.09 [58] and SoapDenovo [59] with a range of kmer lengths (57–64) the final assembly being based on assembly size, number of contigs and contig size compared to *M. paratuberculosis* K10 (Accession number AE016958).

### Genome analysis

Annotation of the *M. paratuberculosis* 43525 genome was performed using the Rapid Annotation and Subsystem Technology (RAST) web application server [60].

Proteinortho was used to conduct comparative blastp searches and clustering analyses [30, 61], which were visualised using Fripan (http://www.vicbioinformatics.com/software.fripan.shtml). Single Nucleotide Polymorphisms (SNPs) were called and used to infer phylogeny using the program CSI Phylogeny 1.0a (https://cge.cbs.dtu.dk/services/CSIPhylogeny/). The parameters for SNP calling were: Minimum depth 10x, minimum relative depth > 10 %, minimum distance between SNPs > 10 bp, minimum SNP quality = 30, read mapping quality score > 25 and minimum Z score 1.96. The phylogenetic tree was imported to FigTree (http://tree.bio.ed.ac.uk/software/figtree/) for visualisation.

Probable orthologues in *M. paratuberculosis* 43525 for PE, PPE and *mce* genes were defined using both the BLASTp algorthrim and Hmmer3 (http://hmmer.janelia.org/) [62]. Orthologues with > 70 % amino acid identity and over 50 % of the sequence length compared to public sequences of the MAC complex and *M. tuberculosis* were considered. Protein databases such as the PFAM database were also used for comparative purposes [63].

In order to investigate the mycobactin cluster of *M. paratuberculosis* 43525 the annotated genome was uploaded to Version 2.0 of the antiSMASH (Antibiotics and Secondary Metabolite Analysis SHell) program [64]. The antiSMASH algorithm identifies backbone enzymes, usually polyketide synthase (PKS), nonribosomal synthetase (NRPS), hybrid PKS-NRPS, or NRPS-like enzymes. Adjacent genes are scanned for the presence of common secondary metabolite gene domains and boundaries are predicted for each cluster. The clusters were then manually analysed and synteny of the mycobactin cluster was visually evaluated by examining whether a gene had orthologues in other mycobacterial species.

Given that the *mbtE* gene of *M. paratuberculosis* 43525 was found to be different to other mycobacterial species. PCR primers *mbtE* fwd (5′ gttacttccccgtcgatccc) and *mbtE* rev (5′ gtagtagagctcccccacca) were designed to amplify the region of *mbtE* that differed from the equivalent gene in *M. paratuberculosis* K10. Automated sequencing to identify PCR products was carried out using the PRISM BigDye™ cycle sequencing system v3.1 and ABI 3730 capillary sequencer (Applied Biosystems).

The *mce* and mycobactin cluster genes were compared across MAC and *M. tuberculosis* with emphasis on members of the MAC complex that infect animals; *M. paratuberculosis* K10 (bovine), *M. avium* ATCC 25291 (avian), *M. paratuberculosis* S397 (sheep) and those that infect humans; *M. paratuberculosis* 43525, *M. avium* 104, *M. avium* TH135. The PE/PPE genes were compared to defined PE/PPE genes from completed genomes only.

### Ethics statement

Ethics approval was not required for this study. All experiments were conducted according to the regulations of the University of New South Wales.

## Availability of supporting data

All supporting data for this article are included as additional files.

## Additional file

Additional file 1: The amino acid percent homology between the PE and PPE genes of *M. paratuberculosis* 43525 compared to other MAC species. (XLSX 15 kb)
